# *Ferula communis* leaf extract: antioxidant capacity, UHPLC–MS/MS analysis, and *in vivo* and *in silico* toxicity investigations

**DOI:** 10.3389/fchem.2024.1485463

**Published:** 2025-01-24

**Authors:** Imad Ed-Dahmani, Mohamed El Fadili, Ghizlane Nouioura, Fahd Kandsi, Yassine El Atki, Hatem A. Abuelizz, Raffaele Conte, Fatima Zahra Lafdil, Abdeslam Taleb, Abdelfattah Abdellaoui, Mustapha Taleb

**Affiliations:** ^1^ Laboratory of Engineering, Electrochemistry, Modelling and Environment, Sidi Mohamed Ben Abdellah University, Faculty of Sciences Fez, Fez, Morocco; ^2^ LIMAS Laboratory, Faculty of Sciences Dhar El Mehraz, Sidi Mohammed Ben Abdellah University, Fez, Morocco; ^3^ Laboratory of Natural Substances, Pharmacology, Environment, Modeling, Health and Quality of Life (SNAMOPEQ), Faculty of Sciences Dhar El-Mehraz, Sidi Mohamed Ben Abdellah University, Fez, Morocco; ^4^ Laboratory of Bioresources, Biotechnology, Ethnopharmacology and Health, Faculty of Sciences, Mohammed First University, Oujda, Morocco; ^5^ High Institute of Nursing Professions and Health Techniques Errachidia, Errachidia, Morocco; ^6^ Department of Pharmaceutical Chemistry, College of Pharmacy, King Saud University, Riyadh, Saudi Arabia; ^7^ Research Institute on Terrestrial Ecosystems (IRET)-CNR, Naples, Italy; ^8^ Laboratory of Water and Environmental Engineering, Hassan II University of Casablanca, Casablanca, Morocco

**Keywords:** *Ferula communis*, flavonoids, polyphenols, 2,2-diphenylpicryl hydroxyl, antioxidant power, docking

## Abstract

**Introduction:**

*Ferula communis* has demonstrated an abundance of pharmacological and antioxidative qualities.

**Methods:**

This study investigates the antioxidant activity of *F. communis* leaf aqueous extract, total polyphenol and flavonoid concentrations, and ultra-high-performance liquid chromatography (UHPLC) composition and then evaluates the toxicity of the plant’s leaves *in vitro* and *in silico*. The major compound of the studied extract, namely, p-hydroxybenzoic acid, was chosen for a molecular docking technique to discover the inhibition mechanism toward antioxidant proteins. In addition, a detailed molecular dynamics simulation was carried out to examine the thermodynamic stability of the produced intermolecular interactions. The antioxidant capacity of the extracts of *F. communis* was evaluated using 2,2-diphenylpicryl hydroxyl (DPPH) radical and ferric reducing antioxidant power (FRAP) procedures. Acute toxicity was tested on albino mice at doses of 200, 300, and 400 mg/kg.

**Results:**

The results show that the polyphenol and flavonoid contents of the extract are significant (0.257 ± 0.003 mg Eq AG/mg and 0.32 ± 0.04 mg Eq Q/mg, respectively). The antioxidant activity illustrates that the extracts have notable activity in DPPH and FRAP assays. The toxicity study revealed that the mice’s behavior, body weight, and organ weights (liver and kidneys) were unaffected by *Ferula communis* leaf extract administration compared to controls. UHPLC–tandem mass spectrometry (MS/MS) analysis of the extract highlights the presence of 11 compounds, the most abundant of which is p-hydroxybenzoic acid, representing 53.65%. The predicted pharmacokinetic characteristics of absorption, distribution, metabolism, excretion, and toxicity (ADMET) attest to the well-absorbed nature of the isolated compounds, with human intestinal absorption (HIA) varying from 42% for arbutin (M3) to 100% for ursolic acid (M4).

**Conclusion:**

In conclusion, the leaves of *Ferula communis* are a good source of natural antioxidants and phenolic compounds. Thus, this study demonstrates that this plant has a wide range of applications, including natural food preservatives, pharmaceuticals, and cosmetics, as evidenced by ongoing research.

## 1 Introduction

Superoxide anion (O, *-), hydrogen peroxide (H_2_O_2_), and hydroxyl radical (HO*) are examples of reactive oxygen intermediates that can damage proteins, nucleic acids, and cell membranes. These intermediates are the source of oxidative stress. Growing evidence shows that reactive oxygen species damage accumulates over time and contributes to many diseases (Aruoma, 1998). On the other hand, the human body’s natural antioxidant mechanism keeps the intracellular redox potential stable (Amin and Bano, 2018). However, natural antioxidants are insufficient in severe or ongoing oxidative stress (Anokwuru et al., 2011).

Historically, medicinal plants have played a significant role in human medicine. Due to poverty and limited access to modern medical treatment, the World Health Organization (WHO) estimates that 65%–80% of people living in underdeveloped nations primarily rely on traditional herbal medicines for their primary medical needs (Muthu et al., 2006). In addition, plants have been the primary source of medicines from ancient times, and all human communities have essentially used plants as sustenance and remedies for various illnesses. Numerous phytochemicals present in plants can improve organ function, act as antioxidants, and supply vital minerals, all of which can dramatically reduce the risk of a wide range of diseases (Azam et al., 2014; Lfitat et al., 2023; ed-dahmani et al., 2024a). In Morocco, especially in the mountainous regions, the low incomes of the local people, geographic isolation, and limited access to primary oral healthcare services contribute to the prevalence of traditional oral healthcare practices, specifically those that use plants to treat oral disorders (Najem et al., 2019). Several studies carried out on traditional herbal treatments have reported toxicity or interaction problems that can lead to therapeutic failures or accidents (Hmamouchi, 1998).

With over 170 species, the genus Ferula is a member of the Apiaceae family. From northern Africa, these are generated westward to central Asia (Watson et al., 2010). Six Ferula species have been observed in Morocco, namely, *Ferula communis*, *Ferula cossoniana*, *Ferula gouliminensis*, *Ferula sauvagei*, *Ferula atlantica*, and *Ferula tingitana*, with more or less extensive distribution areas. The most widespread species, *F. communis,* is very polymorphic and occupies the whole territory except for the desert and arid regions (Alaoui-Faris and Cauwet-Marc, 2006). *F. communis* is well known for its gum resin (l-fāsūẖ), which is extracted from the rootstock through incision and stripping (Al-Yahya et al., 1998). The fruit of *F. communis* is mostly consumed as a vegetable after being steamed or mashed and then diced and seasoned with salt, pepper, and olive oil (Farhood, 2022). A herd usually avoids *F. communis* grazing since it can be poisonous, except for droughts (Nouioura et al., 2024a).

Numerous varieties of Ferula have been employed as herbal anthelmintics and aphrodisiacs, treatments for gastrointestinal diseases (asthma, bronchitis, etc.), and spasmolytic, anti-flatulence, and antidiarrheal medicines (Slinkard and Singleton, 1977b). In addition, *F. communis* has long been used to treat rheumatism, dermatological disorders, trauma, and diabetes (Ed-dahmani et al., 2024a; Ed-Dahmani et al., 2024b). The rhizomes of this plant are used locally for the traditional remedy of skin infections, while the roasted flower buds are used against fever and dysentery (Al-Yahya et al., 1998). The various mechanisms through which Ferula plants mediate their therapeutic effects include inducing apoptosis; inhibiting lipoxygenase, cyclooxygenase (COX), and inducible nitric oxide synthase (iNOS); lowering the levels of prostaglandin E2 (PGE2) and nitric oxide (NO); modifying heat shock protein 70 (Hsp70); and reducing tumor necrosis factor (TNF)-α and interleukin (IL)-6 (Moosavi et al., 2015). The indigenous traditional healers employed various portions of medicinal plants as medicine. Of all the plant components, leaves were most commonly used to treat illnesses, followed by whole plant parts, fruit, stem, root, root bark, seed, flower, and latex (Muthu et al., 2006). Earlier phytochemical investigations on the fruits by high-performance liquid chromatography equipped with a diode-array detector (HPLC–DAD) identified 15 compounds in giant fennel extract, with p-coumaric acid, 3-hydroxybenzoic acid, sinapic acid, and syringic acid being dominant (Nouioura et al., 2024a). The main constituents in the root extract of *F. communis* by ultra-high-performance liquid chromatography (UHPLC)–tandem mass spectrometry (MS/MS) were identified as luteolin (21.48%), vanillic acid (10.98%), and kaempferol (24.57%) (Ed-dahmani et al., 2024a).

Not much research has been conducted on the molecular docking, antioxidant activity, and toxicological activity of *Ferula communis* leaf aqueous extract. This work aims to study the antioxidant activity, total polyphenol, flavonoid content, and UHPLC composition of an aqueous extract of *F. communis* and examine their toxicity impact *in vitro* and *in silico*.

## 2 Materials and methods

### 2.1 Reagents and standards

2,2-Diphenylpicryl hydroxyl (DPPH) radical, butylated hydroxyl toluene (BHT), aluminum chloride (AlC_l3_), quercetin, rutin, gallic acid, iron III chloride (FeC_l3_), potassium ferricyanide (K_3_Fe (CN)_6_), sodium carbonate (Na_2_CO_3_), sodium nitrite (NaNO_2_), and Folin–Ciocalteu reagent were purchased from Sigma-Aldrich (St. Louis, MO, United States). All the other chemicals and solvents used were of analytical grade.

### 2.2 Materials from plants

The *Ferula communis* plant was harvested at its mature age in February 2022 from Taounate city in Morocco. Professor Abdelfattah Abdellaoui, a botanist from the Biology Department, Faculty of Science, University of Sidi Mohamed Ben Abdellah, Fez, Morocco, identified the plant material. A herbarium specimen with voucher number 2299/4-16-1/taw was deposited.

### 2.3 Constructing extracts

The leaves of *Ferula communis* were dried at room temperature (between 20°C and 25°C). For a full day, 18 g of powdered leaves were macerated in 150 mL of distilled water (we have used water as it is traditionally used in preparation methods in Morocco). The resultant macerate was filtered and concentrated using a rotating evaporator for dehydration below space at 40°C. The extracted materials were stored in sterilized Eppendorf sample tubes at 4°C in preparation for later use.

### 2.4 Total contents of flavonoids

The total flavonoid content of the aqueous extract (AE) was ascertained by colorimetric analysis using aluminum chloride. A measure of 500 μL of aluminum chloride (20%) was mixed with 500 µL of the sample or quercetin, following a 1-h reaction under darkness at room temperature (14.0°C ± 2.00°C). Absorbance was read at 420 nm. The total flavonoid amount was presented as milligrams of quercetin equivalents (mg QE/g Dw) for each gram of dry weight of the extract. The calibration curve was constructed using quercetin as the standard (Kara et al., 2022). The phytochemical composition of a plant is under complex control and is affected by both external environmental factors and endogenous circadian rhythms. The environmental factors that directly affect phytochemical profiles and concentrations vary across time of day and time of year (Liebelt et al., 2019).

### 2.5 Total phenolic content

The Folin–Ciocalteu method (Slinkard and Singleton, 1977a) was used to determine the total polyphenol concentrations of the aqueous extracts. A known extract dilution of 0.5 mL and a sodium carbonate solution of 7% were combined with 2.5 mL of 10% Folin–Ciocalteu, following a 2-h reaction in darkness at ambient temperature (14.00°C ± 2.00°C). At 760 nm, absorbance was measured. Milligrams of gallic acid equivalents (mg GAE/g Dw) were used to express the extract’s total phenol concentration in grams of dry weight. Gallic acid served as the norm in the calibration curve’s building.

### 2.6 LC–MS/MS analysis of the *Ferula communis* aqueous extract

The chemical profile of the *Ferula communis* extract was established using UHPLC coupled with high-resolution mass spectrometry (LCMS-8060, Shimadzu Italy, Milan). Specifically, the source settings were configured as follows: a nebulizing gas flow rate of 2.9 L/min, a heating gas flow rate of 10 L/min, an interface temperature of 300°C, a linear ion trap (LIT) detector temperature of 250°C, a thermal block temperature of 400°C, and a drying gas flow rate of 10 L/min. LC–MS detection was set in the negative ionization mode. We developed an internal database that includes polyphenol derivatives through qualitative analysis. The separation of compounds and standards was carried out on a C18 column with dimensions of 3 × 100 mm and a particle size of 2.6 µm (Phenomenex, Torrance, CA, United States). The elution of the extract components was achieved under isocratic conditions using acetonitrile (A) and water containing 0.01% formic acid (B), with a total run time of 25 min. The mobile phase comprised acetonitrile (A) and water with 0.01% formic acid (B). The *Ferula communis* extract was added to a mixture of acetonitrile and water in a 1:1 ratio. The solution (20 µL) was then diluted with acetonitrile (980 µL) and injected into the instrument for analysis. A molecule was considered positive if its area under the curve was greater than that of the blank sample. In cases of very similar structures, the distinction was made using retention time, with the instrument configured to record the molecular mass in the third quadrupole (Kandsi et al., 2021).

### 2.7 Antioxidant activity

#### 2.7.1 DPPH assay for free radical scavenging

Applying Hui-Chun Wu’s (Wu et al., 2003) technique, the antioxidant activity of *Ferula communis* extract was tested to scavenge the DPPH radical. A measure of 0.1 mL of the sample or standard at various concentrations was mixed with 1.5 mL of the methanol extract containing 0.1 mmol of DPPH. Following a half-hour incubation period in the shadows at a comfortable temperature (14.0°C ± 2.00°C), the mixture’s absorbance at 517 nm was determined. BHT was used as a positive control. The following formula was used to get the % inhibition:

$$I\;\left(\text{\%}\right)=\left(1\;‐\;\left(\text{As}/A_{0}\right)\right)\times 100,$$

where As is the sample’s absorbance and A0 is the absorbance of the negative control.

#### 2.7.2 Ferric reducing antioxidant power

We investigated the *Ferula communis* extract’s iron-reducing antioxidant power using the Oyaizu method (Oyaizu, 1986). A measure of 200 μL of the extract is mixed with 500 μL of potassium ferricyanide (K_3_Fe (CN)_6_) 1% and 500 µL of phosphate buffer (0.2 M, pH 6). After incubating the solution obtained at 50°C for 20 min, 500 μL of trichloroacetic acid (TCA) (10%) was added to the mixture and centrifuged at 3,000 rpm for 10 min. The upper layer of the solution (2.5 mL) was mixed with 500 µL of distilled water and 100 µL of FeCl_3_ (0.1%). Quercetin was utilized as a norm when detecting absorbances at 700 nm. The outcome was given as an EC_50_ value (mg/mL). Plotting the absorbance against the corresponding extract concentration allowed for calculating the extract concentration (EC_50_) corresponding to 0.5 absorbances.

### 2.8 Toxicity study

#### 2.8.1 Animal materials

A total of 20 Swiss albino mice (10 male and 10 female) were used for the experimental study of acute toxicity tests. The mice weighed 20–34 g and were 4–6 weeks old. The animals were placed in specially equipped rooms, with regulated lighting from 6 a.m. to 6 p.m. and a temperature of 25°C ± 2°C. This research was carried out following the guidelines for using and caring for laboratory animals established by the Faculty of Science Ethics Committee in Fez, Morocco (USMBA-SNAMOPEQ 2017-03).

#### 2.8.2 Acute toxicity

Acute toxicity was accomplished following the procedure outlined by Costa-Silva et al. (2008) in accordance with Guideline No. 423; the mice were divided into four batches of five individuals each and adapted for 3 days before the initial test. They were kept on an empty stomach for 18 h before administering the different doses. The first batch (control) received distilled water, while the second, third, and fourth groups were orally administered a one-time gavage of *F. communis* root extract at respective doses of 200, 300, and 400 mg/kg bw. Over 14 days, daily assessments of their general behavior and body weight were carried out. On day 14, all mice were anesthetized and sacrificed, and the weight of their organs (liver and kidneys) was measured. The blood sample was taken to carry out the biochemical analysis.

### 2.9 In silico study

In the present work, the chemical composition of *Ferula communis* leaf was examined using *in silico* predictions of physicochemical and ADME-Tox pharmacokinetic features, followed by the molecular docking simulation for p-hydroxybenzoic acid (M8) as the major compound of the studied extract, which was complexed to NADPH oxidase protein to explore the chemical interactions mode that could provide a rationale for the antioxidant activity of *Ferula communis* leaf (Assaggaf et al., 2023; Benkhaira et al., 2023; Jeddi et al., 2023). Initially, pkCSM and SwissADME servers were properly used to predict 11 chemical compounds’ physicochemical properties and absorption, distribution, metabolism, excretion, and toxicity (ADMET) characteristics (El fadili et al., 2023a, El fadili et al., 2023b; Abechi et al., 2024; Er-rahmani et al., 2024; Nouioura et al., 2024e). Second, AutoDock software was equally used to explore the inhibition mechanism with corresponding binding energies in kcal/mol (Nouioura et al., 2024a; Nouioura et al., 2024d; Nouioura et al., 2024c), in which the targeted protein coded in the protein data bank by 2CDU.pdb was prepared by adding the Gasteiger charges and removing all suspended ligands bound to the targeted protein (El fadili et al., 2022b, El fadili et al., 2022a, El fadili et al., 2023c, El fadili et al., 2024). Finally, Discovery Studio 2021 software was also employed to visualize the produced intermolecular interactions in two and three dimensions (Bouzammit et al., 2024c; Bouzammit et al., 2024b; Bouzammit et al., 2024a). The thermodynamic stability of the produced interactions was equally investigated by the molecular dynamics technique using the Desmond program, a package of Schrodinger software (Er-rajy et al., 2023), in which the output file of molecular docking was used as an input file of molecular dynamics (El fadili et al., 2023d).

## 3 Statistical analysis

The means of the three experiments were expressed using the standard deviation (SD) and standard error (SEM). Variances were checked for normality and homogeneity to identify the type of statistical study (parametric or non-parametric). The mean difference’s significance was verified using variance analysis (one-way and two-way ANOVA). Tukey’s tests were performed using Prism by GraphPad 8.0.2 at p = 0.05.

## 4 Results and discussion

### 4.1 Total polyphenol and flavonoid contents

The polyphenolic compounds and their antioxidant activity are crucial factors in assessing the samples’ antioxidant capability. Utilizing the Folin–Ciocalteu and aluminum chloride colorimetry techniques, respectively, to ascertain the total phenolic and total flavonoid contents, Table 1 presents the results of the investigations. The outcomes indicate that the extract of *F. communis* leaves has a high level of flavonoid content (0.32 ± 0.04 mg eq AG/mg) and total polyphenol (0.257 ± 0.003 mg eq AG/mg). When comparing our research to other studies, our results were in line with those of Aydin et al. (2021), who used an ethanol–water (50:50) extract.

**TABLE 1:** Total polyphenol and flavonoid content of *Ferula communis* leaves’ extracts.

	Polyphenol content (mg eq AG/mg)	Flavonoid content (mg eq AG/mg)
Aqueous extract	0.257 ± 0.003	0.32 ± 0.04

Each value represents the means ± SD of the three experiments.

In Nouioura et al. (2024a), *F. communis* fruits were macerated with various solvents, including methanol, ethanol, water, hexane, acetone, ethyl acetate, and chloroform, which were used individually. The ethanol extract exhibited the highest total polyphenol content (62.20 ± 0.11 mg GAE/g DW), followed by the methanol extracts (60.82 ± 0.32 mg GAE/g DW) and aqueous extracts (44.04 ± 0.22 mg GAE/g DW), respectively. The flavonoid content exhibited the highest extraction with the ethanol extract (17.09 mg QE/g DW), while the aqueous extract showed lower extraction yields with 9.31 ± 0.24 and 8.97 ± 0.47 mg GAE/g DW. In a previous study, *F. communis* roots (Ed-dahmani et al., 2024d) were tested with distilled water. The results show that the extract contains 0.194 ± 0.004 mg polyphenols, expressed as GA equivalent/mg, and 0.820 ± 0.031 mg flavonoids, expressed as GA equivalent/mg.

These high quantities of polyphenols and flavonoids make the plant a powerful antioxidant. Numerous investigations have verified the affirmative correlation between high levels of phenol and flavonoids, robust overall antioxidant potential, and the ability to mitigate free radical damage (Sarikurkcu et al., 2018; Tangjitjaroenkun, 2018).

### 4.2 LC–MS/MS examination of the *Ferula communis* extract

**FIGURE 1:**
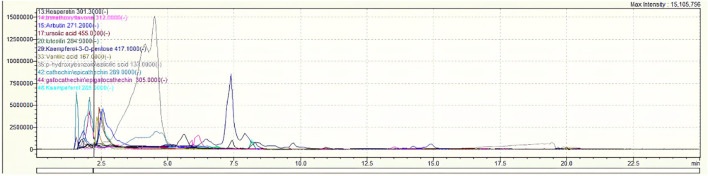
UHPLC chromatograms of the aqueous extract of *Ferula communis*.

The results we present are from an analysis conducted by LC–MS-MS, revealing the composition of the aqueous extract of *Ferula communis*. This analysis highlights the presence of 11 compounds, the most abundant of which is p-hydroxybenzoic/salicylic acid, representing 53.65%. The main molecular families within this extract are flavonoids, glucosides, triterpenoids, and phenolic acids. These conclusions are supported by the empirical data provided in Table 2. Additionally, Figure 1 represents the chromatogram, further underscoring the critical importance of the aqueous extract in preserving and enriching these compounds.

**TABLE 2:** Phytochemical composition of the aqueous extract of *Ferula communis* revealed by LC–MS/MS.

Compound	Formula	Classes of compounds	(M-H) ^-^	AUC	% AUC
Hesperetin	C_16_H_14_O_6_	Flavonoid	301,300	30.864.912	2.02%
Trimethoxyflavone	C_18_H_16_O_5_	Flavonoid	3,120,000	28,631,550	1.87%
Arbutin	C_12_H_16_O_7_	Glucosidic	2,712,000	115.934.449	7.59%
Ursolic acid	C_30_H_48_O_3_	Triterpenoid	4,550,000	67.861.417	4.44%
Luteolin	C_15_H_10_O_6_	Flavonoid	2,849,000	71.673.895	4.69%
Kaempferol-3-O-pentoside	C_20_H_18_O_10_	Flavonoid	4,171,000	114.690.785	7.51%
Vanillic acid	C_8_H_8_O_4_	Phenolic acid	1,670,000	44.742.570	2.93%
P-hydroxybenzoic\salicylic acid	C_7_H_6_O_3_	Phenolic acid	1,370,000	819.040.964	53.65%
Catechin\epicatechin	C_15_H_14_O_6_	Flavonoid	289.0000	94.509.137	6.19%
Gallocatechin\epigallocatechin	C_15_H_14_O_7_	Flavonoid	305.0000	62.493.779	4.09%
Kaempferol	C_15_H_10_O_6_	Flavonoid	285.0000	76.097.990	4.98%

We had no information on the phenolic composition of the aqueous extract of *F. communis* leaves until now. This study represents the first qualitative and quantitative analysis of the leaf polyphenols of this plant, conducted using UHPLC. In total, 11 compounds have been identified in the aqueous extract of *F. communis* leaves. These include hesperetin, trimethoxyflavone, arbutin, ursolic acid, luteolin, kaempferol-3-O-pentoside, vanillic acid, p-hydroxybenzoic acid (or salicylic acid), catechin (or epicatechin), gallocatechin (or epigallocatechin), and kaempferol (Table 2). The fruits of the same plant exhibit 15 phenolic compounds, among which gallic acid, caffeic acid, catechin, 4-hydroxybenzoic acid, catechin hydrate, succinic acid, syringic acid, 3-hydroxybenzoic acid, naringin, cinnamic acid, ferulic acid, p-coumaric acid, sinapic acid, quercetin 3-O-β-D-glucoside, and rutin were identified and quantified using HPLC-DAD (Nouioura et al., 2024a). In addition, in Al-Yahya et al. (1998), various phenolic compounds such as resorcinol, ferulic acid, syringic acid, and coumarin were identified as the predominant components in the fruits of *F. communis*. Another study conducted by Rahali et al. (2019) using RP-HPLC revealed that methanolic extracts from the flowers, fruits, and stems of *F. communis* collected from northern Tunisia were primarily composed of resorcinol, ferulic acid, syringic acid, and coumarin.

### 4.3 Antioxidant properties of the extract from *Ferula communis* leaves

Several authors link phenolic chemicals, particularly the secondary metabolites of plants, to a range of biological characteristics. Because of this, disclosing such substances has evolved into an essential initial step toward ideal utilization. The antioxidant activity of *Ferula communis* extract leaves was evaluated using FRAP and DPPH techniques (Table 3).

**TABLE 3:** Antioxidant activity of leaves of *F. communis* extracts with DPPH and FRAP tests.

DPPH IC_50_ (mg/mL)		FRAP EC_50_ (mg/mL)	
	BHT	Extract	Quercetin
0.263 ± 0.008^a^	0.118 ± 0.0001^b^	15.659 ± 0.087^a^	0.033 ± 0.0004^b^

Each value represents the means ± SD of the three experiments (ANOVA I and Tukey tests at p < 0.05).

The lowest IC_50_ value (concentration capable of inhibiting 50% of DPPH) indicated the highest level of antioxidant activity. In light of this research’s results, the inhibitory power of the aqueous extract of *Ferula communis* leaves is greater, with an IC_50_ value of the order of 0.263 ± 0.008 mg/mL. The activity level remains under the requirement for BHT (0.118 ± 0.0001 mg/mL) to be used as the reference.

Table 3 presents the FRAP assay results. The extracts studied show that the aqueous leaf extract of *Ferula communis* has a remarkable reducing power (EC_50_ = 15.659 ± 0.087 mg/mL). However, its power is still less than the reference value of quercetin (EC_50_ = 0.033 mg/mL).

DPPH^●^ is a radical widely used in model systems to investigate the neutralizing capabilities of various natural materials, such as anthocyanins, phenolic compounds, and pure plant extracts (Chang et al., 2007). Antioxidants can produce stable free radicals by neutralizing the free radical oxidation cycle, preventing further oxidation. Furthermore, antioxidants can scavenge DPPH radicals by donating hydrogen, resulting in the DPPH radical discoloring when extracts at increasing concentrations are added, creating decreased DPPH-H (Burits and Bucar, 2000).

Radical reactions are ubiquitous in all organisms and play a more or less direct role in gene modification, reproduction, and disease defense (Guillouty, 2016). Based on four different ways of action, antioxidant activity has been assessed. It is crucial to conduct a variety of assays to take the chemical makeup of the extract into account, as it acts through several methods. Variations in the way phenolic compounds react to the various antioxidant reaction mechanisms and the variety of resulting products. It was also noted that a good resulting product of these reactions could explain the differences in correlations found using applicable antioxidant tests (El Atki et al., 2019). Since most phenolic compounds are hydrophilic molecules, their low viscosity and smaller density allow for easier diffusion in polar to semi-polar solvents (Fernanda et al., 2016; Taroq et al., 2018).

According to published research, the FRAP approach is sensitive for determining the overall antioxidant power of fresh biological fluids, including medicinal plant compounds and plant homogenates (Szollosi and Szollosi Varga, 2002; Rattanachitthawat et al., 2010). The FRAP assay is used to examine the potential effects of medicinal plants by measuring their overall antioxidant power (Szollosi and Szollosi Varga, 2002).

When comparing our study with others on antioxidant activity using the DPPH method and the FRAP technique, Rahali et al. (2019) examined the antioxidant activity of the methanolic extract from the flowers, fruits, and stem of *Ferula communis*. The analysis revealed that the stem extract had the lowest scavenging activity (IC_50_ = 168 mg/mL) by the DPPH method.

Ed-Dahmani et al. (2024c) assessed the antioxidant activity of the fruits of the *Ferula communis* plant by DPPH and FRAP. Extracts were prepared by maceration with methanol and distilled water. The results showed that the inhibitory power by DPPH of the methanol extracts of fruit (IC_50_ = 0.076 ± 0.039 mg/mL) is greater compared to the aqueous extracts (IC_50_ = 0.26 ± 0.006 mg/m). The FRAP test showed that the methanol extract of fruit (IC_50_ = 202.71 ± 1.471 mg/mL) has greater antioxidant power than aqueous extracts (IC_50_ = 208.04 ± 5.69 mg/mL). Ed-dahmani et al. (2024b) tested *F. communis* roots by the DPPH method. The extract exerted a considerable scavenging action on DPPH, with an IC_50_ value of 0.820 ± 0.031 mg/mL.

### 4.4 Acute toxicity

The acute toxicity of the *Ferula communis* leaf aqueous extract was assessed by measuring its effects on the body weight, organ weight, and general behavior of mice.

#### 4.4.1 Effects of extract leaves of *Ferula communis* on the general behavior of mice

Table 4 illustrates the single oral administration of the *Ferula communis* leaves extract in mice at different doses (200, 300, and 400 mg/kg). After 14 days, no poisoning symptoms (death, coma, urination, change in skin, mobility, sleep, vigilance, vomiting, tremors, aggression, diarrhea, and eye color) have been noticed.

**TABLE 4:** Effect of the extract on albino mice’s overall behavior.

Observation	Control	Doses		
		200 mg/kg	300 mg/kg	400 mg/kg
Death	Alive	Alive	Alive	Alive
Coma	Not observed	Not observed	Not observed	Not observed
Mobility	Normal	Normal	Normal	Normal
Aggressiveness	Normal	Normal	Normal	Normal
Urination	Normal	Normal	Normal	Normal
Change in skin	No effect	No effect	No effect	No effect
Tremor	Normal	Normal	Normal	Normal
Sleep	Normal	Normal	Normal	Normal
Vomiting	Normal	Normal	Normal	Normal
Diarrhea	Normal	Normal	Normal	Normal
Vigilance	Normal	Normal	Normal	Normal
Eye color	No effect	No effect	No effect	No effect

#### 4.4.2 Mouse body effects of acute intoxication with the *Ferula communis* leaves extract

**FIGURE 2:**
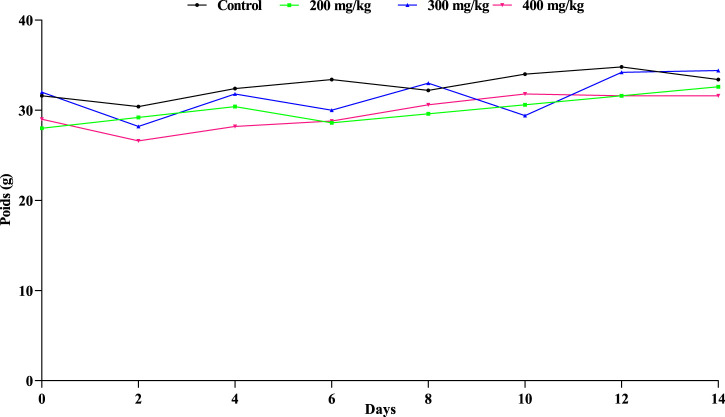
Extract’s effect on mice’s weight oscillations. A non-significant difference at p < 0.05 is indicated by the letter (a) in the means (±SD, n = 3) (ANOVA I and Tukey’s tests).

As demonstrated in Figure 2, each mouse remained relatively stable compared to the mice receiving the same control, despite the weight variations observed in those receiving different dosages.

#### 4.4.3 *Ferula communis* leaf extract’s effects on mice organs

**FIGURE 3:**
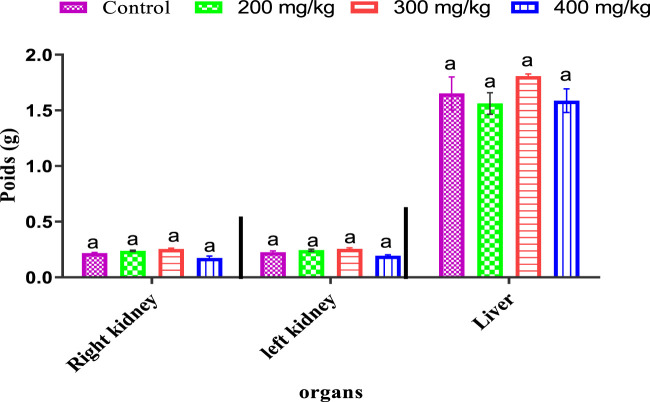
Variations in the treated and control animals’ respective organ weights. A non-significant difference at p < 0.05 among all treatments using one-way ANOVA, followed by the Tukey’s test, is indicated by the letter (a) in the means (±SD, n = 3).

The results show that, when comparing the mice given the doses of *Ferula communis* leaf extract to the mice used as controls, there was no significant variation in the relative weights of the major organs (liver and kidneys) (Figure 3).

Plants classified as toxic include chemical compounds or active principles that, when ingested, inhaled, or touched by humans or animals, can result in harm, illness, or even death (Serrano, 2018). Toxicological studies are necessary to detect the range of doses utilized in animal experiments and acquire scientific data concerning medical problems and the consequences of these products. Animals’ changes in body weight are a key sign of their overall health (Sakai and Tamashiro, 2005). According to the results of the acute toxicity study, animals given a single dosage of 200, 300, or 400 mg/kg did not exhibit any deaths or notable signs of intoxication (such as vomiting, tremors, sleeplessness, aggression, diarrhea, mobility, eye color, urination, coma, and death) compared to the control group. Because there was no discernible change in the mice’s body weight between the treated groups and the controls following a single gavage, it seems probable that oral administration of plant extracts from *Ferula communis* leaves does not affect normal mouse growth.

When there are disturbances in the metabolism of proteins, carbs, or lipids, loss of appetite often leads to weight loss (Ezeonwumelu et al., 2024) (Chokshi, 2007; Tahraoui et al., 2010). In addition, the aqueous extract of the leaves of the *Ferula communis* plant did not affect the kidneys and the liver weights of the mice that were administered the extract. In toxicological studies, the relative weight of the organs is considered a reasonably sensible indicator (Lüllmann-Rauch, 2008). Furthermore, there has been no appreciable shift in the weight of the liver or reins, suggesting that the application of diluted extracts from *Ferula communis* leaves has not interfered with their regular development. These results imply that this extract is not lethal to mice and that they tolerate it well.

### 4.5 Biochemical parameters

Toxicity studies are performed on mice to estimate the toxicity of the plant and the right dose to determine the health risks associated with plants for use by humans. Since alterations in the blood system have a greater predictive value for human toxicity, hematological indices in animals are crucial for assessing the toxicity risk (Adinortey et al., 2012).

#### 4.5.1 Hematological parameters

Table 5 provides a summary of the acute toxicity of *Ferula communis* plant leaves in aqueous extract on different hematological indicators. The parameters tested were total red blood cells (RBCs), hemoglobin (HGB), HCT (hematocrit), mean corpuscular hemoglobin concentration (MCHC), and platelets (PLT) (Table 5). As illustrated in Table 5, for HGB, HCT, and MCHC, no appreciable change was found between the mice treated with the different doses (200, 300, and 400 mg/kg) and the mice used as the control. However, compared to the control group, we observed a significant (p < 0.05) decrease in red blood cells (RBCs) and MCHC at 400 mg/kg. In comparison with the control group, mice that were administered different doses showed a non-significant (p < 0.05) decrease in hemoglobin (HGB). Platelets increased at the 400 mg/kg dose compared to the treated mice and those used as the reference.

**TABLE 5:** Effect of administering mice an oral *Ferula communis* extract on specific hematological indicators.

Parameter	RBC (10^6^/μL)	HGB (g/dL)	HCT (%)	MCV (fL)	MCHC (pg)	PLT (10^5^/μL)
Control	7.57 ± 0.29	14.12 ± 0.16	45.18 ± 0.69	60.42 ± 3.71	17.58 ± 0.57	6.18 ± 0.49
200 mg/kg	7.42 ± 0.23	13.55 ± 1.55	45.65 ± 2.38	60.73 ± 1.24	18.43 ± 2.13	6.83 ± 2.24
300 mg/kg	7.66 ± 0.15	14.26 ± 1.38	45.28 ± 2.63	61.38 ± 2.67^*^	18.24 ± 2.43	7.46 ± 1.06
400 mg/kg	6.97 ± 1.07^*^	14.75 ± 2.21	45.25 ± 3.19	58.55 ± 3.29^*^	18.07 ± 1.58	8.36 ± 2.87^**^

At *p < 0.05, **p < 0.01, and ***p < 0.001, the comparison between the control and other groups is shown by the mean ± SEM for each value (ANOVA I and Tukey’s tests).

Olson et al. (2000) stated that there is a link between animal and human toxicity regarding detrimental effects on the gastrointestinal tract, cardiovascular system, and hematological system (Oslon et al., 2000). In tissue and blood, alkaline phosphatase, alanine, and aspartate aminotransferases are significant enzymes used to measure cell death, cytosolic activity, and cell membrane integrity (Akanji et al., 1993).

Hepatocytes are responsible for detoxifying toxins, whether they originate from the external sources or from within the body. At the same time, the kidney is responsible for cleansing the blood and removing waste products (Ozer et al., 2008). Since the liver and kidneys play essential roles in an organism’s survival, it is crucial to analyze their function when assessing the toxicity of medications and plant extracts (Leticia Acosta Wolf, 1972).

#### 4.5.2 Serum biochemical parameters

**FIGURE 4:**
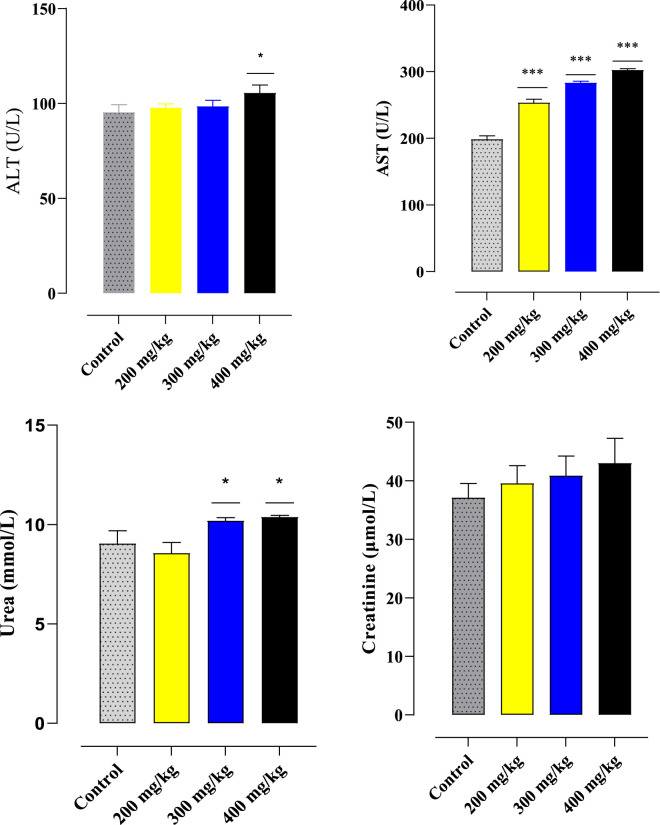
Effects of particular biochemical markers of oral administration of the *F. communis* extract on mice. At *p < 0.05, the comparison between the control and other groups is shown by the mean ± SEM for each value (ANOVA I and Tukey’s tests).

The biochemical characteristics (aspartate aminotransferase (AST), creatinine, urea, and alanine aminotransferase (ALT)) of injected and untreated Souris are listed in Figure 4. The current results clearly show that the oral administration of aqueous leaf extracts from the plant *Ferula communis* does not appear to result in any significant changes in creatinine in the mice treated with different doses compared to the control mice. The aminotransferase (ALT) levels did not show any significant effect at doses of 200 mg/kg and 300 mg/kg, but a significant effect was observed at 400 mg/kg. The aminotransferase (AST) levels showed a significant effect at all doses. Urea levels were affected at doses of 300 and 400 mg/kg.

### 4.6 Correlation

The current investigation examined the correlations between polyphenol content, flavonoid content, and antioxidant activity in the leaves and fruits of *F. communis* (Nouioura et al., 2024a; Ed-Dahmani et al., 2024b).

The results (Table 6) showed that the correlation test is considered an excellent tool for revealing relationships between different parameters studied, with a good correlation between flavonoids and polyphenols (0.981) and between FRAP and polyphenol (0.951). However, the correlation between FRAP and DPPH is weak (−0.491).

**TABLE 6:** Pearson correlation coefficients.

	Polyphenol	Flavonoid	DPPH	FRAP
Polyphenol	1	0.981***	−0,73,572**	0,951,274***
Flavonoid		1	−0,85,188***	087,455***
DPPH			1	−0,49,104
FRAP				1

Statistical significance, *p< 0.05; **p< 0.01; ***p < 0.001.

Nouioura et al. (2024a) assessed the antimicrobial properties of *F. communis* extracts in ethanol (EtOH), acetone (AcE), or water (AqE) against four bacterial strains, namely, *Escherichia coli, Bacillus subtilis, Proteus mirabilis*, and *Staphylococcus aureus*, as well as four fungal strains, namely, *Fusarium oxysporum*, *Aspergillus niger*, *Aspergillus flavus*, and *Candida albicans.*

The AcE extract displayed the highest inhibitory activity against *P. mirabilis* with an inhibition diameter of 19.00 ± 1.00 mm and an MIC value of 2.50 ± 0.00 mg/mL, followed by *E. coli* with 11.50 ± 1.50 mm of inhibition zone and an MIC value of 2.50 ± 0.00 mg/mL. The extract also exhibited inhibitions of 11.00 and 9.00 mm against *S. aureus* and *B. subtilis*, respectively.

The EtOH extract exhibited the greatest activity against *E. coli*, with an IC_50_ inhibition diameter of 14.00 ± 1.00 mm and an MIC value of 0.312 ± 0.00 mg/mL, and the smallest inhibition zone of 9.00 ± 0.00 mm was observed for *S. aureus*. The antifungal activities of the *F. communis* extract against C. *albicans*, *A. niger*, A. *flavus*, and *F. oxysporum* were compared to that of the fungicide fluconazole. AcE exhibited significant activity against *F. oxysporum*, with a percent inhibition of 20.6% ± 1.4% and an MIC value of 5.0 ± 0.0 mg/mL.

In a previous study, the toxicity of the fruits of the *Ferula communis* plant was assessed using the hydroethanolic solvent. A single oral administration of hydroethanol at doses of 200, 300, and 400 mg/kg to mice showed no sign of toxicity (mobility, aggressiveness, vigilance, tremors, sleep, vomiting, or diarrhea), and no deaths occurred in the treated mice at the tested doses (Ed-Dahmani et al., 2024a). In another study, the toxicity of the *F. communis* roots was studied with distilled water. When given orally to mice at doses of 200, 300, and 400 mg/kg, *F. communis* roots did not induce any toxicity (mobility, aggressiveness, vigilance, tremors, sleep, vomiting, or diarrhea) or death in the treated animals at the tested doses (Ed-dahmani and Mssillou, 2024a).

### 4.7 Physicochemical, ADME-Tox pharmacokinetics, and molecular docking

**FIGURE 5:**
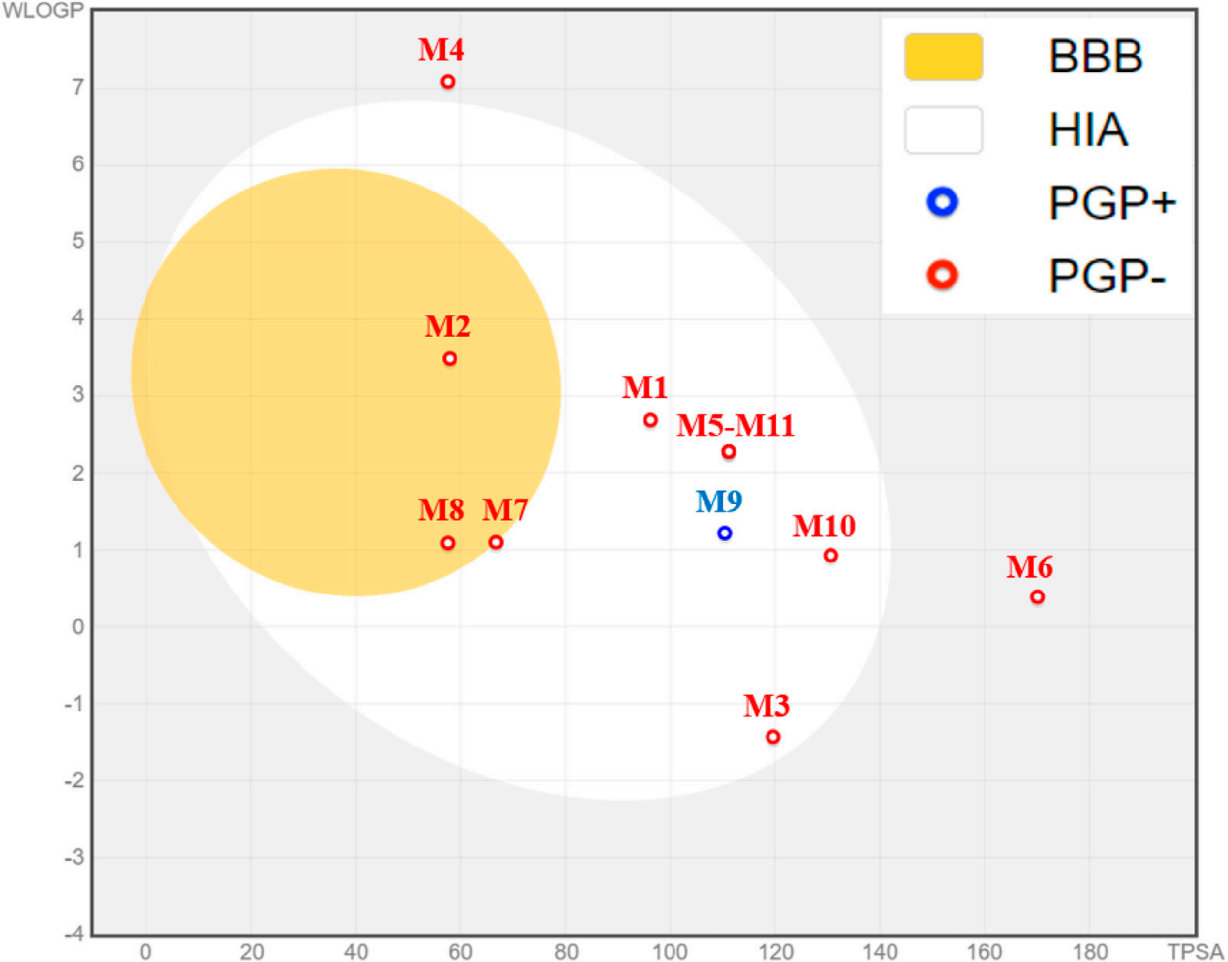
Predictive model of Egan’s boiled egg for 11 extracted molecules.

The prediction of physicochemical properties reveals that all extracted molecules from the *Ferula communis* leaf meet all five rules of Lipinski (Aloui et al., 2024; Seddoqi et al., 2024), where the number of acceptors and donors of hydrogen bonds does not exceed ten and five, respectively. The molecular weights are less than 500 g/mol. The molar refractivity index falls within the range defined by (Kara et al. (2022) [130], and the lipophilicity in the octanol/water solvent is defined by a LogP value of less than 5, as presented in Table 7. Moreover, the prediction of the pharmacokinetic properties of ADMET confirms that extracted molecules are well-absorbed, with human intestinal absorption (HIA) ranging from 42% for arbutin (M3) to 100% for ursolic acid (M4); these molecules show good permeability to the central nervous system (CNS) and blood–brain barrier (BBB). The metabolism test also shows that M2 and M4 molecules are considered substrates of 3A4 cytochrome, and the chemical compounds labeled M2, M5, and M11 were predicted as potent agents to inhibit 1A2, 2C9, 2C19, and 3A4 cytochromes. The AMES toxicity test declares the safety of all extracted molecules except for M6, M9, and M10 molecules. However, all extracted molecules from the *Ferula communis* leaf are safe from any skin sensitization and hepatotoxicity effects except for M4, as presented in Table 8. The predictive model of Egan declares that M2, M7, and M8 are part of the yellow-boiled egg, so they are predicted to cross the BBB with the highest probability. M1, M3, M5, M9, M10, and M11 are part of the white Egan’s egg, so they were predicted to be passively absorbed by the gastrointestinal tract, as displayed in Figure 5.

**TABLE 7:** Prediction of physicochemical properties of 11 chemical compounds extracted from *Ferula communis* leaf based on Lipinski’s rule of five.

Molecule Number	Physicochemical properties					Lipinski rules
	Molecular weight (g/mol)	Molar refractive index	Log P (octanol/water)	Hydrogen bond acceptors	Hydrogen bond donors	Categorical (Yes/No)
Rule	≤500	40≤ MR ≤ 130	<5	≤10	<5	
M1	302.28	77.83	2.51	6	3	Yes
M2	312.32	87.40	2.96	5	0	Yes
M3	272.25	62.61	1.64	7	5	Yes
M4	456.70	136.91	3.95	3	2	Yes
M5	286.24	76.01	1.86	6	4	Yes
M6	418.35	102.17	1.76	10	6	Yes
M7	168.15	41.92	1.40	4	2	Yes
M8	138.12	35.42	0.85	3	2	Yes
M9	290.27	74.33	1.47	6	5	Yes
M10	306.27	76.36	1.37	7	6	Yes
M11	286.24	76.01	1.70	6	4	Yes

M1, hesperetin; M2, trimethoxyflavone; M3, arbutin; M4, ursolic acid; M5, luteolin; M6, kaempferol-3-O-pentoside; M7, vanillic acid; M8, p-hydroxybenzoic; M9, epicatechin; M10, gallocatechin; M11, kaempferol.

**TABLE 8:** Prediction of ADME and toxicity pharmacokinetic properties of 11 compounds extracted from *Ferula communis* leaves.

Molecule Number	Absorption	Distribution		*Metabolism*							*Excretion*	*Toxicity*		
	Human intestinal absorption	Blood–brain barrier permeability	Central nervous system permeability	Substrate		Inhibitor					Total clearance	AMES test of toxicity	Hepatotoxicity	Skin sensitization
				Cytochromes										
				2D-6	3A-4	1A-2	2C-19	2C-9	2D-6	3A-4				
	(% absorbed)	(Log BB)	(Log PS)	(No/Yes)							Numeric (log ml/min/kg)	(No/Yes)		
M1	78.513	−0.952	−3.356	No	No	No	No	No	No	No	0.473	No	No	No
M2	97.687	0.446	−2.24	No	Yes	Yes	Yes	Yes	No	No	0.385	No	No	No
M3	42.175	−0.865	−4.338	No	No	No	No	No	No	No	0.595	No	No	No
M4	100	−0.137	−1.117	No	Yes	No	No	No	No	No	0.083	No	Yes	No
M5	84.159	−1.152	−2.455	No	No	Yes	No	No	No	Yes	0.615	No	No	No
M6	56.687	−1.498	−4.339	No	No	No	No	No	No	No	0.601	Yes	No	No
M7	82.827	−0.417	−2.559	No	No	No	No	No	No	No	0.625	No	No	No
M8	74.377	−0.331	−2.91	No	No	No	No	No	No	No	0.666	No	No	No
M9	72.519	−1.066	−3.395	No	No	No	No	No	No	No	0.266	Yes	No	No
M10	65.43	−1.385	−3.721	No	No	No	No	No	No	No	0.474	Yes	No	No
M11	75.342	−1.234	−2.368	No	No	Yes	No	No	No	No	0.592	No	No	No

M1, hesperetin; M2, trimethoxyflavone; M3, arbutin; M4, ursolic acid; M5, luteolin; M6, kaempferol-3-O-pentoside; M7, vanillic acid; M8, p-hydroxybenzoic; M9, epicatechin; M10, gallocatechin; M11, kaempferol.

**FIGURE 6:**
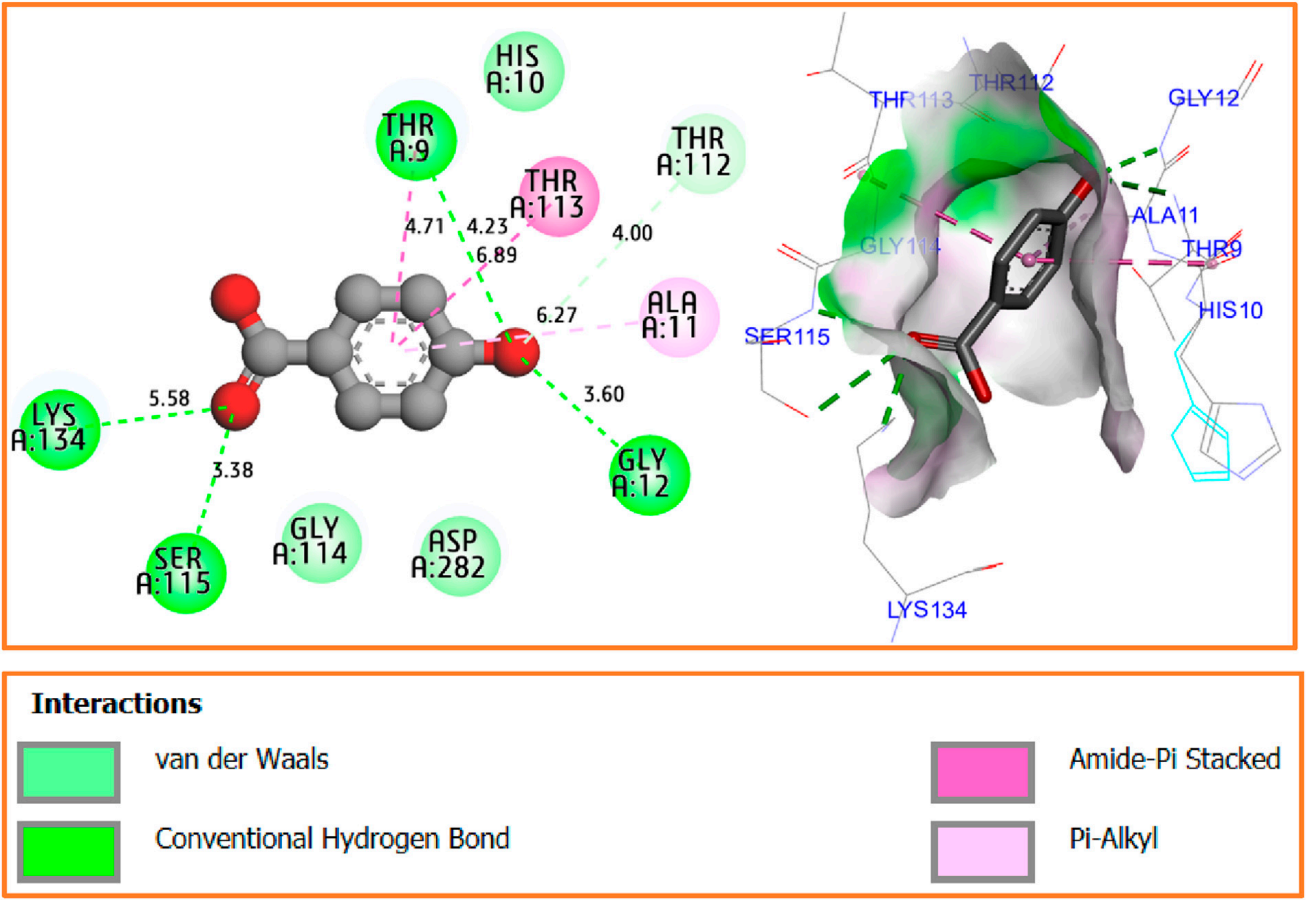
Two- and three-dimensional views of produced intermolecular interactions for p-hydroxybenzoic acid in complex with the NADPH oxidase protein (2CDU.pdb).

The results of molecular docking simulation confirm that the major compound of *Ferula communis* leaf extract, p-hydroxybenzoic acid, was complexed with the NADPH oxidase protein (2CDU.pdb) with the lowest binding energy of −5.21 kcal/mol. This interaction produced four conventional hydrogen bonds with Ser115, Thr9, Gly12, and Lys134 amino acids residues in A chain and one amide–pi-stacked interaction with Thr113 amino acid residue; furthermore, one van der Waals bond was formed with Thr112 amino acid residue, and one Pi–alkyl bond was formed with Ala11 amino acid residue, as displayed in Figure 6.

**FIGURE 7:**
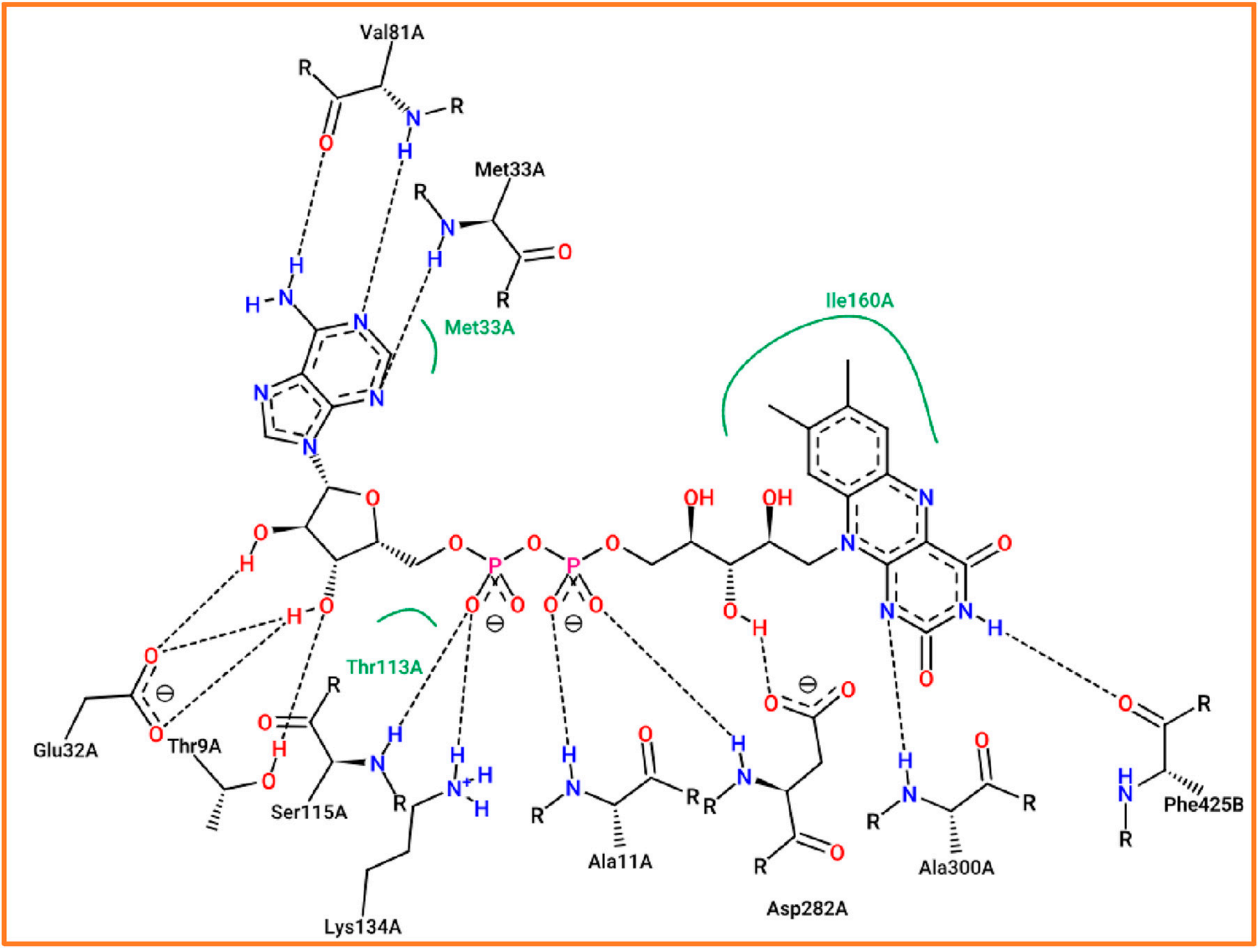
Active sites of the NADPH oxidase protein (2CDU.pdb) in complex with flavin adenine dinucleotide co-crystallized ligand.

The process of molecular docking is well-validated as the studied compound was docked to various active sites of the antioxidant protein, including Ser115, Thr9, Lys134, and Ala11 amino acid residues, which were the same active sites in the same chain that were obtained by the flavin adenine dinucleotide co-crystalized ligand, as presented in Figure 7.

#### 4.7.1 Molecular dynamics simulation

**FIGURE 8:**
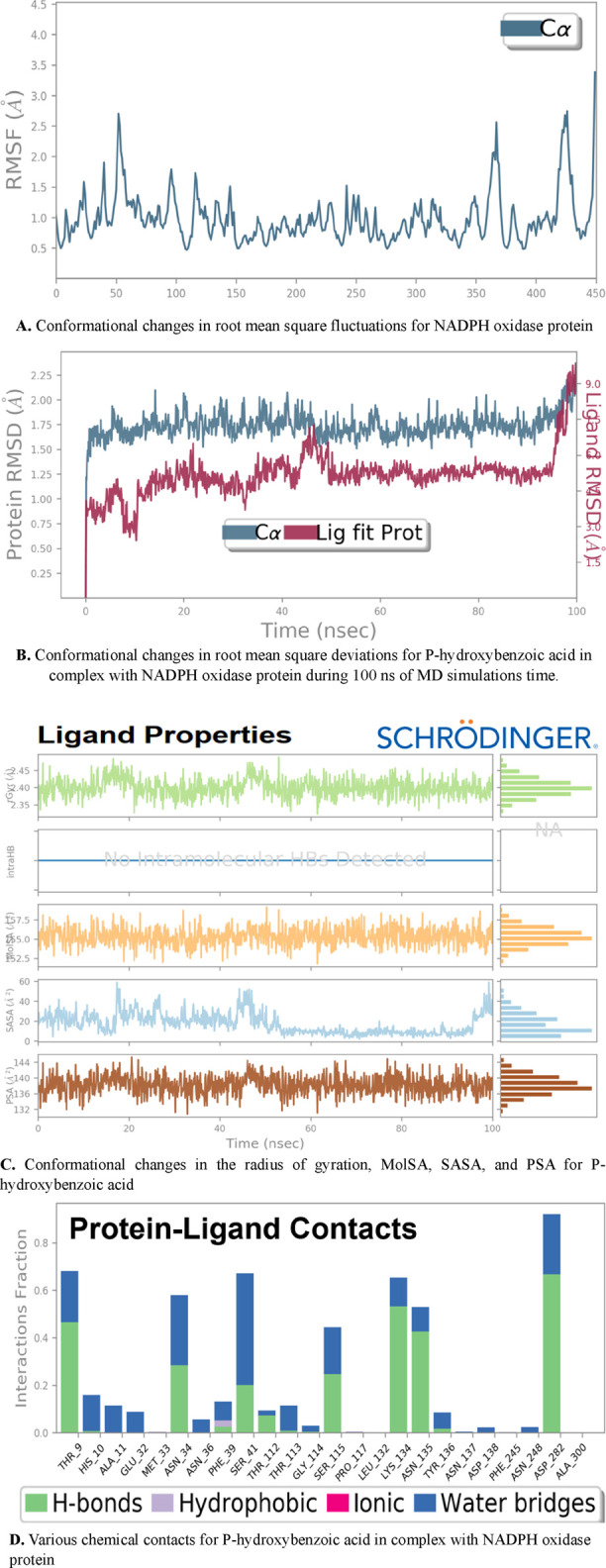
Dynamic changes in RMSF, RMSD, rGyr, MolSA, SASA, PSA, and possible intermolecular contacts for p-hydroxybenzoic acid in complex with NADPH oxidase protein. **(A)** Conformational changes in root mean square fluctuations for NADPH oxidase protein. **(B)** Conformational changes in root mean square deviations for p-hydroxybenzoic acid in complex with NADPH oxidase protein during 100 ns of MD simulations time. **(C)** Conformational changes in the radius of gyration, MolSA, SASA, and PSA for p-hydroxybenzoic acid. **(D)** Various chemical contacts for p-hydroxybenzoic acid in complex with NADPH oxidase protein.

The results of molecular dynamics simulations reveal good levels of molecular stability for the p-hydroxybenzoic acid ligand in complex with the NADPH oxidase protein, confirming the strongest intermolecular interactions previously obtained by molecular docking. The mentioned stability is justified by minimum root-mean-square fluctuations (RMSFs) for the targeted protein that oscillated around equilibrium and did not exceed 3 Å throughout the 100 ns MD simulation time, as presented in Figure 8A. Second, the root mean square deviations (RMSDs) show that the candidate ligand does not diffuse far from the active sites of the NADPH oxidase protein in which the red deviations for the p-hydroxybenzoic ligand change slightly and in parallel with the blue deviations for the protein targets, as shown in Figure 8B. Third, minimal conformational changes were observed in the physicochemical characteristics of p-hydroxybenzoic acid during 100 ns of MD simulation time, with all properties oscillating with negligible fluctuations, as shown in Figure 8C. The radius of gyration (rGyr), which measures the extension of the candidate ligand, is equivalent to its principal moment of inertia; the molecular surface area (MolSA) is an equivalent value of van der Waals surface area, calculated with a probe radius of 1.4 Å; the solvent-accessible surface area (SASA) is the surface area of a molecule accessible by a water molecule; and polar surface area (PSA) is the solvent-accessible surface area of a molecule to which only oxygen and nitrogen atoms contribute. Finally, Figure 8D also confirms that hydrogen bonds in green with water bridges in blue have a more significant interaction fraction than other types of ligand–protein contacts, particularly those detected toward amino acid residues Ser115, Thr9, and Lys134, as the active sites of the protein NADPH oxidase.

## 5 Conclusion

The purpose of this work is to investigate the antioxidant activity, flavonoid concentration, total polyphenol, and UHPLC composition of the *F. communis* aqueous extract. Furthermore, we aim to examine its toxicity effects both *in vitro* and *in silico*.

The analysis of the *F. communis* leaf extract highlights the presence of 11 compounds, with p-hydroxybenzoic/salicylic acid being the most abundant, representing 53.65%. The extract shows remarkable polyphenol and flavonoid content, along with exceptional antioxidant activity, as assessed by the DPPH and FRAP methods. The plant demonstrated no sign of toxicity, and the pharmacokinetic parameters calculated using ADMET illustrated that ursolic acid (M4) has a 100% HIA and arbutin (M3) shows a 42% change in HIA, indicating good absorption of the extracted molecules. In light of this result, *F. communis* leaves can be utilized as a source of phenol and antioxidants.

## Data Availability

The original contributions presented in the study are publicly available. This data can be found here: https://zenodo.org/records/14720182.
