# Supplementary material for: Ferula communis leaf extract: antioxidant capacity, UHPLC–MS/MS analysis, and in vivo and in silico toxicity investigations
Source: Front Chem. 2025 Jan 24;12:1485463. doi: 10.3389/fchem.2024.1485463 (PMC11803407; doi:10.3389/fchem.2024.1485463)
Supplement: Supplementary file 1 [file DataSheet2.pdf]

# = Shimadzu LabSolutions Quant. Browser Data Report =

Acquired by	: System Administrator
Data Acquired	: 16/05/2024 21:36:01
Sample Type	: Unknown
Sample Name	: imad 1
Sample ID	:
Sample Amount	: 1
Dilution Factor	: 1
Vial#	: 75
Injection Volume	: 15 uL
Data Filename	: imad 1_052.lcd
Method Filename	: polifenoli screening SIM C18 25 min.lcm
Processed by	: System Administrator
Modified Date	: 19/06/2024 10:14:33

198.9000(+)@1
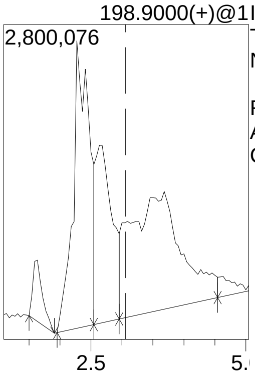

ID#:1 m/z: 198.9000
Type:Target
Name:syringic acid SIM

Ret.Time:0.000
Area:0
Conc.:N.D.(W/B)

168.9000(-)@6
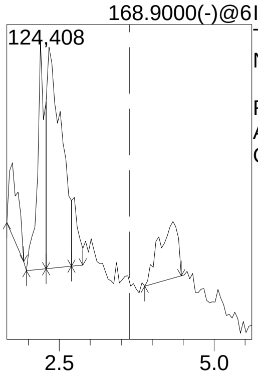

ID#:2 m/z: 168.9000
Type:Target
Name:gallic acid SIM

Ret.Time:0.000
Area:0
Conc.:N.D.(W/B)

301.0000(-)@7
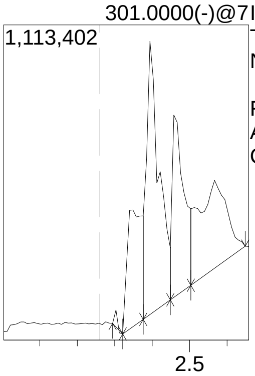

ID#:3 m/z: 301.0000
Type:Target
Name:quercetin sim

Ret.Time:0.000
Area:0
Conc.:N.D.(W/B)

162.9000(-)@8
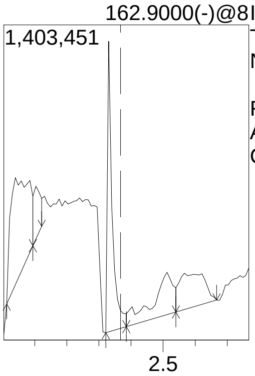

ID#:4 m/z: 162.9000
Type:Target
Name:p coumarci acid SIM

Ret.Time:0.000
Area:0
Conc.:N.D.(W/B)

303.2000(-)@9
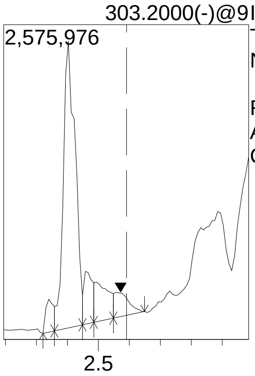

ID#:5 m/z: 303.2000
Type:Target
Name:oleochantal SIM

Ret.Time:2.860
Area:3261113
Conc.:0.000ppm

153.0500(-)@10
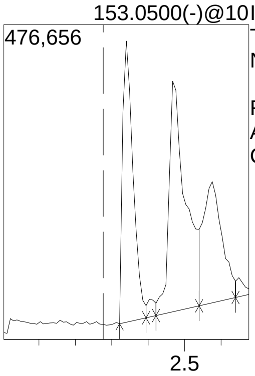

ID#:6 m/z: 153.0500
Type:Target
Name:hydroxytyrosol SIM

Ret.Time:0.000
Area:0
Conc.:N.D.(W/B)

193.0000(-)@11ID#:7 m/z: 193.0000

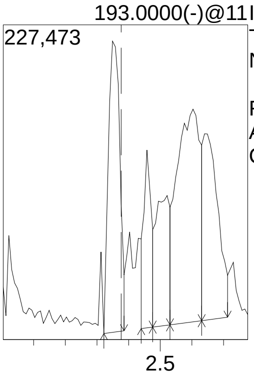

Type:Target
Name:trans ferulic acid SIM

Ret.Time:0.000
Area:0
Conc.:N.D.(W/B)

539.0000(-)@12ID#:8 m/z: 539.0000

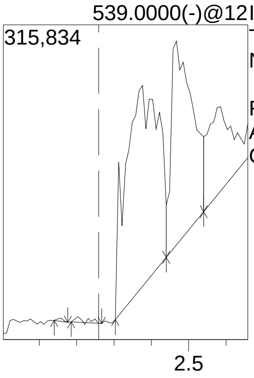

Type:Target
Name:oleuropein SIM

Ret.Time:0.000
Area:0
Conc.:N.D.(W/B)

301.3000(-)@13ID#:9 m/z: 301.3000

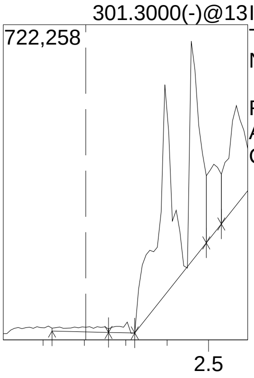

Type:Target
Name:Hesperetin

Ret.Time:0.000
Area:0
Conc.:N.D.(W/B)

312.0000(-)@14ID#:10 m/z: 312.0000

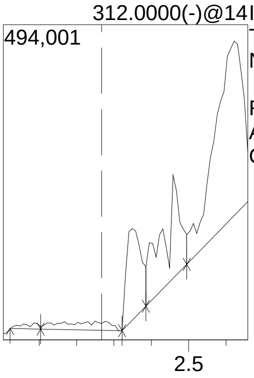

Type:Target
Name:trimethoxyflavone

Ret.Time:0.000
Area:0
Conc.:N.D.(W/B)

271.2000(-)@15ID#:11 m/z: 271.2000

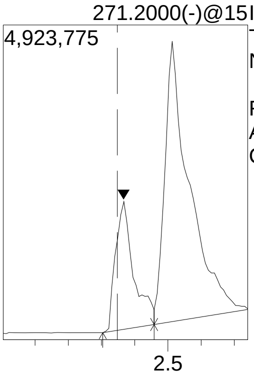

Type:Target
Name:Arbutin

Ret.Time:1.831
Area:38476358
Conc.:0.000ppm

359.0000(-)@16ID#:12 m/z: 359.0000

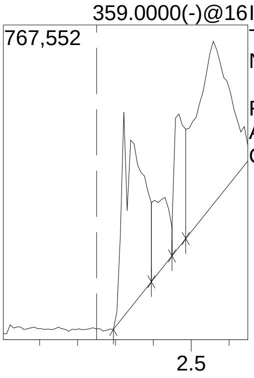

Type:Target
Name:Rosmarinic acid

Ret.Time:0.000
Area:0
Conc.:N.D.(W/B)

455.0000(-)@17ID#:13 m/z: 455.0000

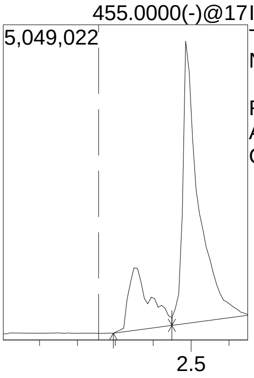

Type:Target
Name:ursolic acid

Ret.Time:0.000
Area:0
Conc.:N.D.(W/B)

269.0000(-)@18ID#:14 m/z: 269.0000

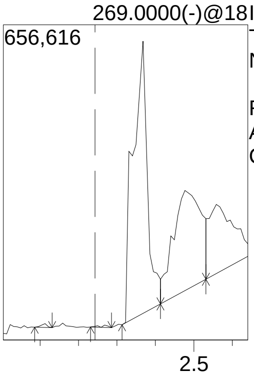

Type:Target
Name:Apigenin

Ret.Time:0.000
Area:0
Conc.:N.D.(W/B)

537.1000(-)@19ID#:15 m/z: 537.1000

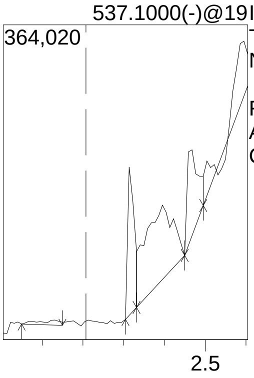

Type:Target
Name:Amentoflavone

Ret.Time:0.000
Area:0
Conc.:N.D.(W/B)

284.9000(-)@20ID#:16 m/z: 284.9000

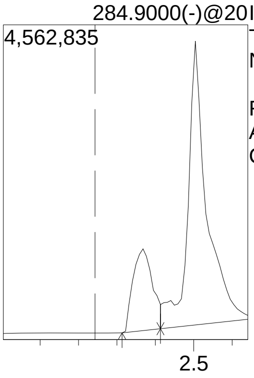

Type:Target
Name:luteoilin

Ret.Time:0.000
Area:0
Conc.:N.D.(W/B)

463.1000(-)@21ID#:17 m/z: 463.1000

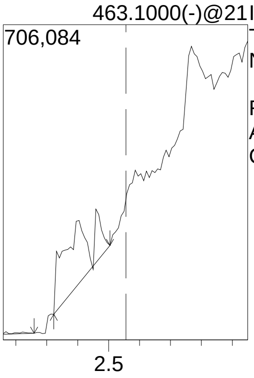

Type:Target
Name:quercetin-3-O-glucoside

Ret.Time:0.000
Area:0
Conc.:N.D.(W/B)

477.0000(-)@22ID#:18 m/z: 477.0000

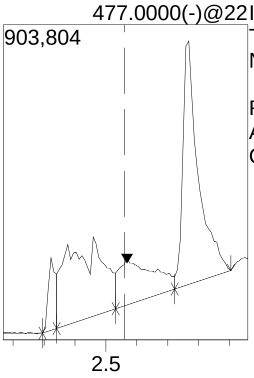

Type:Target
Name:quercetin-3-O- glucuronic acid

Ret.Time:2.842
Area:4904621
Conc.:0.000ppm

609.1000(-)@23ID#:19 m/z: 609.1000

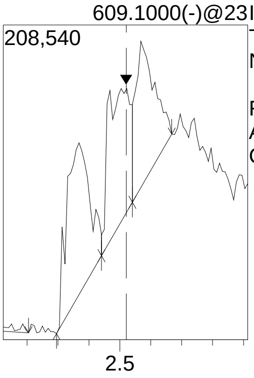

Type:Target
Name:Kaempferol-3-O-glucose

Ret.Time:2.601
Area:2007474
Conc.:0.000ppm

609.1000(-)@24ID#:20 m/z: 609.1000

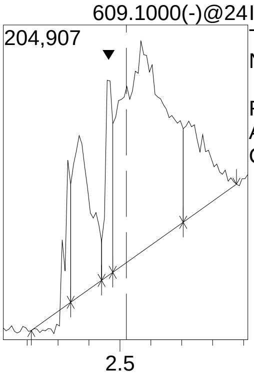

Type:Target
Name:Quercetin-3-O.hexose deoxyhex

Ret.Time:2.317
Area:869696
Conc.:0.000ppm

623.1000(-)@25ID#:21 m/z: 623.1000

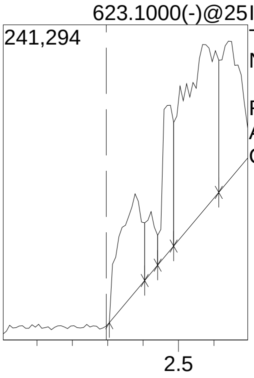

Type:Target
Name:Isorhamnetin- 3-O Rutinoside

Ret.Time:0.000
Area:0
Conc.:N.D.(W/B)

447.1000(-)@26ID#:22 m/z: 447.1000

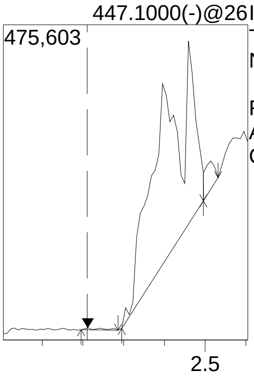

Type:Target
Name:Isorhamnetin-7-O- Pentose

Ret.Time:1.061
Area:40696
Conc.:0.000ppm

447.1000(-)@27

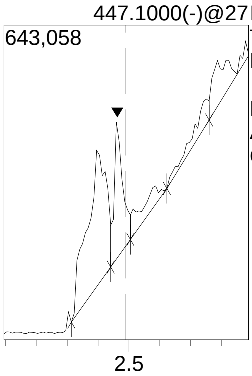

ID#:23 m/z: 447.1000
Type:Target
Name:luteoilin 7-O-glucoside

Ret.Time:2.311
Area:2670182
Conc.:0.000ppm

461.1000(-)@28

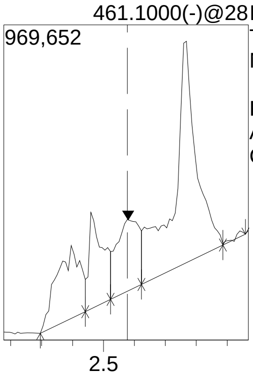

ID#:24 m/z: 461.1000
Type:Target
Name:Kaempferol-3-O-glucuronic acid

Ret.Time:2.900
Area:5646605
Conc.:0.000ppm

417.1000(-)@29

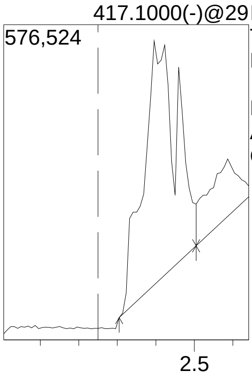

ID#:25 m/z: 417.1000
Type:Target
Name:Kaempferol-3-O-pentose

Ret.Time:0.000
Area:0
Conc.:N.D.(W/B)

593.1000(-)@30

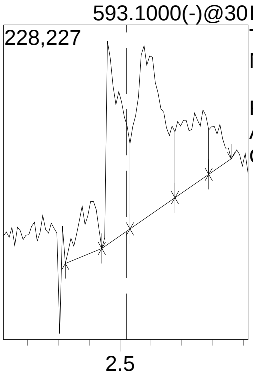

ID#:26 m/z: 593.1000
Type:Target
Name:Kaempferol-3-O-hexose deohyh

Ret.Time:0.000
Area:0
Conc.:N.D.(W/B)

153.4000(-)@31

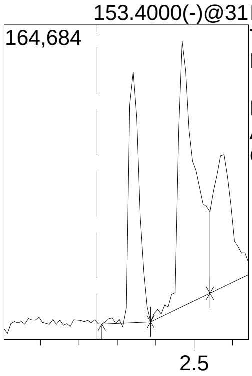

ID#:27 m/z: 153.4000
Type:Target
Name:Tyrosol

Ret.Time:0.000
Area:0
Conc.:N.D.(W/B)

153.0000(-)@32

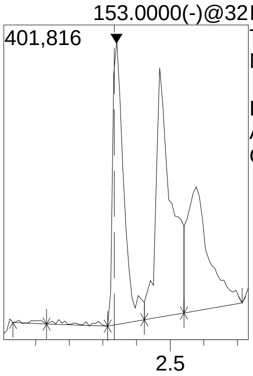

ID#:28 m/z: 153.0000
Type:Target
Name:Protocathecoic acid

Ret.Time:1.701
Area:4099367
Conc.:0.000ppm

167.0000(-)@33

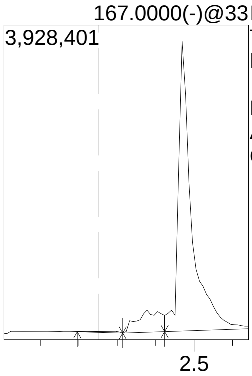

ID#:29 m/z: 167.0000
Type:Target
Name:Vanillic acid

Ret.Time:0.000
Area:0
Conc.:N.D.(W/B)

197.0000(-)@34

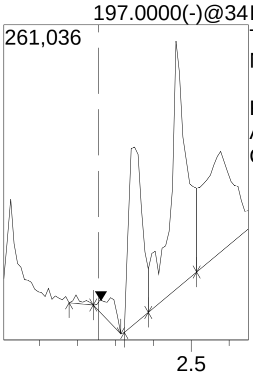

ID#:30 m/z: 197.0000
Type:Target
Name:syringic acid

Ret.Time:1.309
Area:251417
Conc.:0.000ppm

137.0000(-)@35

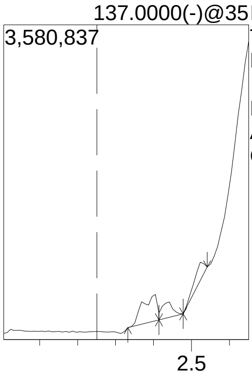

ID#:31 m/z: 137.0000
Type:Target
Name:p-hydroxybenzoic\salicilic acid

Ret.Time:0.000
Area:0
Conc.:N.D.(W/B)

153.0000(-)@36

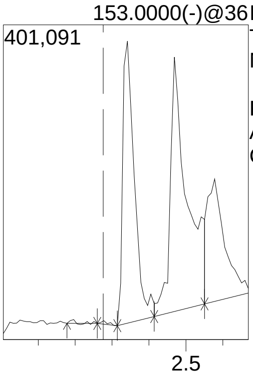

ID#:32 m/z: 153.0000
Type:Target
Name:Gentisic acid

Ret.Time:0.000
Area:0
Conc.:N.D.(W/B)

179.0000(-)@37

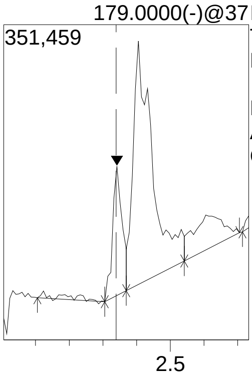

ID#:33 m/z: 179.0000
Type:Target
Name:Caffeic acid

Ret.Time:1.707
Area:1295376
Conc.:0.000ppm

223.0000(-)@38

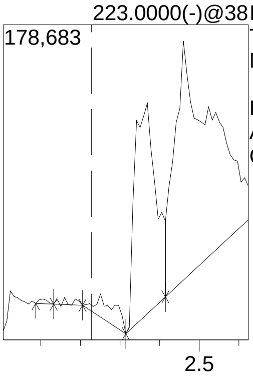

ID#:34 m/z: 223.0000
Type:Target
Name:Sinapic acid

Ret.Time:0.000
Area:0
Conc.:N.D.(W/B)

193.0000(-)@39

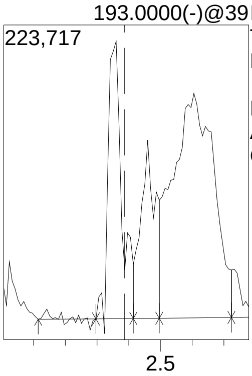

ID#:35 m/z: 193.0000
Type:Target
Name:Ferulic acid

Ret.Time:0.000
Area:0
Conc.:N.D.(W/B)

147.0000(-)@40

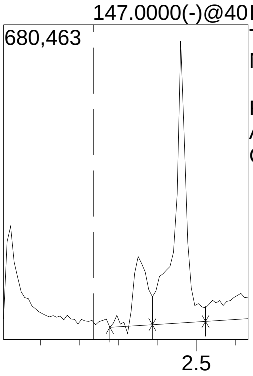

ID#:36 m/z: 147.0000
Type:Target
Name:Trans-cinnamic acid

Ret.Time:0.000
Area:0
Conc.:N.D.(W/B)

353.0000(-)@41

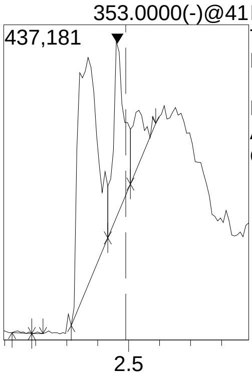

ID#:37 m/z: 353.0000
Type:Target
Name:Chlorogenic acid

Ret.Time:2.319
Area:2785180
Conc.:0.000ppm

289.0000(-)@42

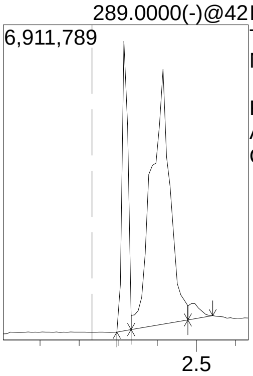

ID#:38 m/z: 289.0000
Type:Target
Name:cathechin\epicathechin

Ret.Time:0.000
Area:0
Conc.:N.D.(W/B)

457.0000(-)@43 ID#:39 m/z: 457.0000

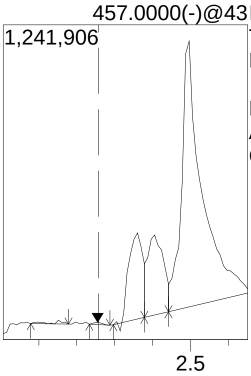

Type:Target
Name:gallocathechin\epigallocathechin

Ret.Time:1.275
Area:67354
Conc.:0.000ppm

305.0000(-)@44 ID#:40 m/z: 305.0000

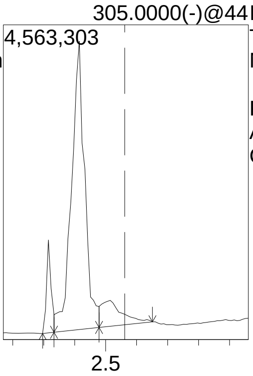

Type:Target
Name:gallocathechin\epigallocathechin

Ret.Time:0.000
Area:0
Conc.:N.D.(W/B)

441.0000(-)@45 ID#:41 m/z: 441.0000

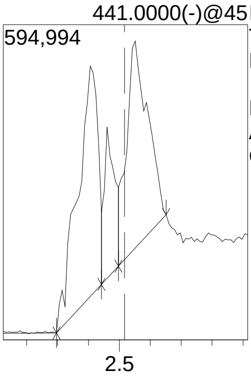

Type:Target
Name:cathechin gallate

Ret.Time:0.000
Area:0
Conc.:N.D.(W/B)

577.0000(-)@46 ID#:42 m/z: 577.0000

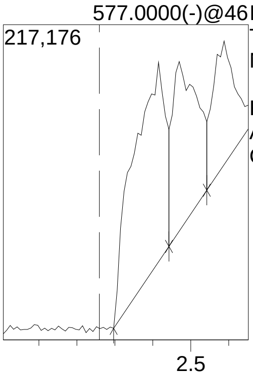

Type:Target
Name:Procianidin

Ret.Time:0.000
Area:0
Conc.:N.D.(W/B)

317.0000(-)@47 ID#:43 m/z: 317.0000

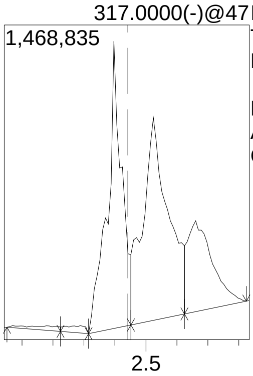

Type:Target
Name:Myricetin

Ret.Time:0.000
Area:0
Conc.:N.D.(W/B)

285.0000(-)@48 ID#:44 m/z: 285.0000

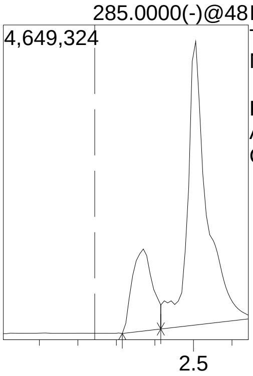

Type:Target
Name:Kaempeferol

Ret.Time:0.000
Area:0
Conc.:N.D.(W/B)

609.0000(-)@49 ID#:45 m/z: 609.0000

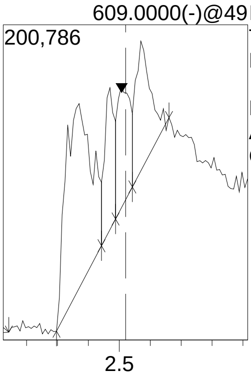

Type:Target
Name:rutin

Ret.Time:2.539
Area:997911
Conc.:0.000ppm

579.0000(-)@50 ID#:46 m/z: 579.0000

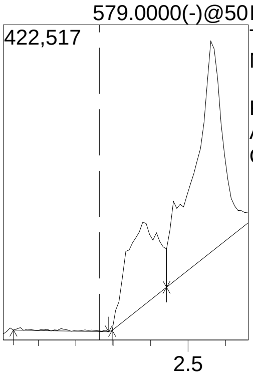

Type:Target
Name:narigin

Ret.Time:0.000
Area:0
Conc.:N.D.(W/B)

536.1000(+)@2ID#:47 m/z: 536.1000

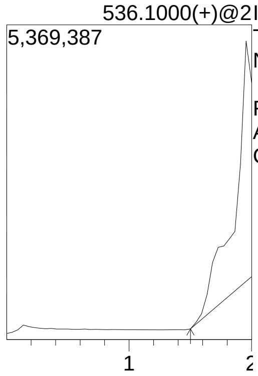

Type:Target
Name:Licopene

Ret.Time:0.000
Area:0
Conc.:N.D.(W/B)

772.8000(+)@3ID#:48 m/z: 772.8000

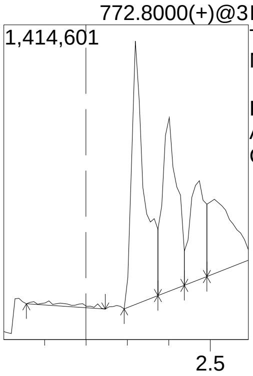

Type:Target
Name:delphinidin3-rutinoside-5-galacto

Ret.Time:0.000
Area:0
Conc.:N.D.(W/B)

465.2000(+)@4ID#:49 m/z: 465.2000

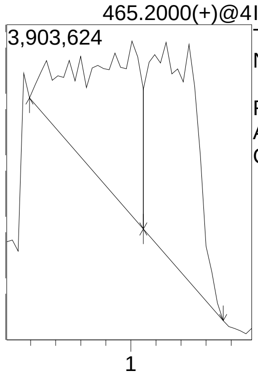

Type:Target
Name:delphinidin-3-glucoside

Ret.Time:0.000
Area:0
Conc.:N.D.(W/B)

611.0000(+)@5ID#:50 m/z: 611.0000

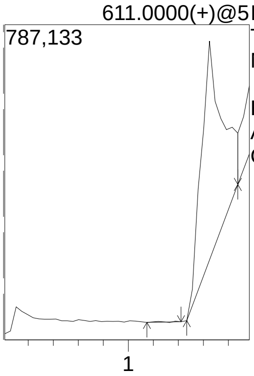

Type:Target
Name:delphinidin-3-rutinoside

Ret.Time:0.000
Area:0
Conc.:N.D.(W/B)

249.0000(-)@51ID#:51 m/z: 249.0000

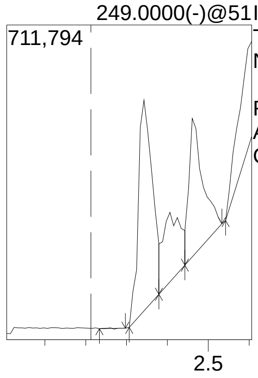

Type:Target
Name:N-caffeoylputrescine

Ret.Time:0.000
Area:0
Conc.:N.D.(W/B)

353.0000(-)@52ID#:52 m/z: 353.0000

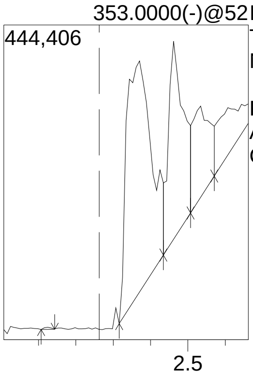

Type:Target
Name:3-caffeoylquinic acid

Ret.Time:0.000
Area:0
Conc.:N.D.(W/B)

470.0000(-)@53ID#:53 m/z: 470.0000

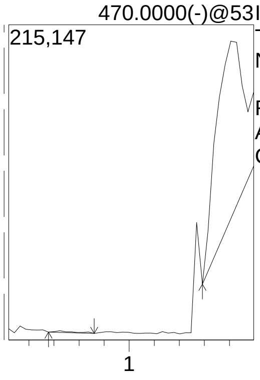

Type:Target
Name:Dihydroxycinnamoyl amide

Ret.Time:0.000
Area:0
Conc.:N.D.(W/B)

468.0000(-)@54ID#:54 m/z: 468.0000

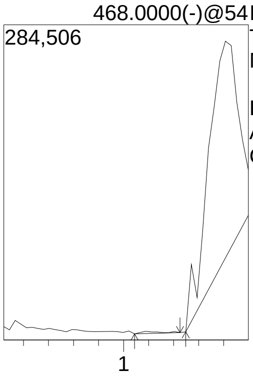

Type:Target
Name:N,N'-dicaffeoylspermidine

Ret.Time:0.000
Area:0
Conc.:N.D.(W/B)
