# Supplementary material for: Ferula communis leaf extract: antioxidant capacity, UHPLC–MS/MS analysis, and in vivo and in silico toxicity investigations
Source: Front Chem. 2025 Jan 24;12:1485463. doi: 10.3389/fchem.2024.1485463 (PMC11803407; doi:10.3389/fchem.2024.1485463)

SHIMADZU LabSolutions

Analysis Report

<Sample Information>

Sample Name	: imad 1		
Sample ID	:		
Data Filename	: imad 1_052.lcd		
Method Filename	: polifenoli screening SIM C18 25 min.lcm		
Batch Filename	: notte.lcb		
Vial #	: 1-75	Sample Type	: Unknown
Injection Volume	: 15 uL		
Date Acquired	: 16/05/2024 21:36:01	Acquired by	: System Administrator
Date Processed	: 19/06/2024 10:14:33	Processed by	: System Administrator

<Chromatogram>

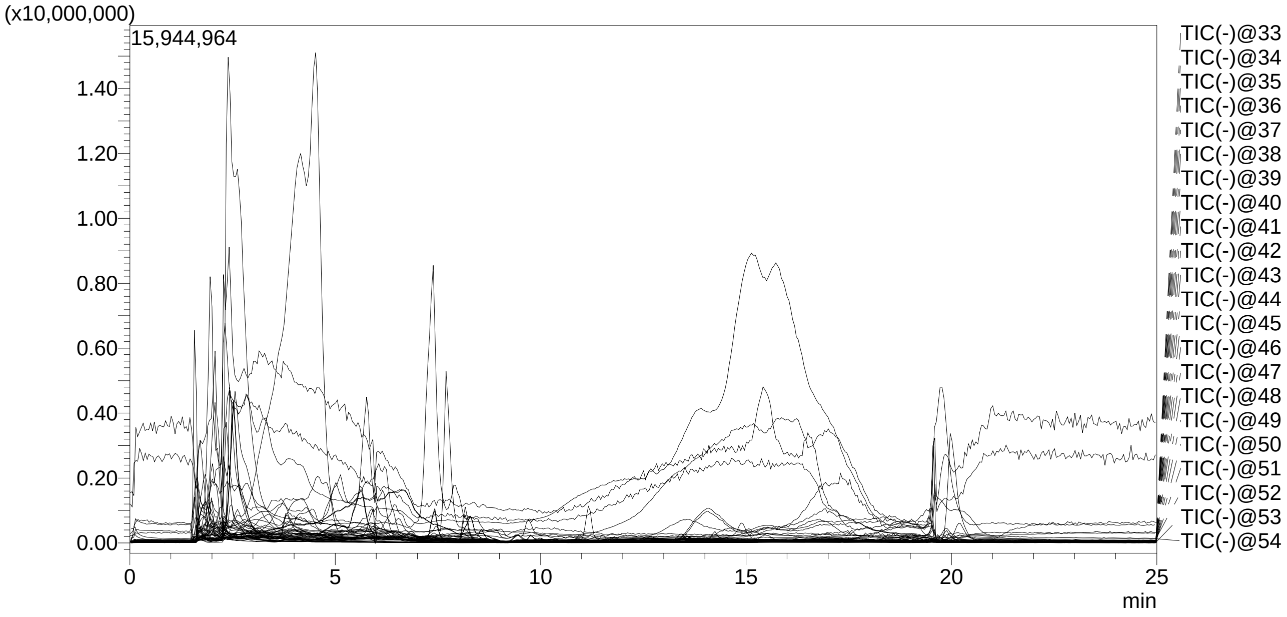

Supplement: Supplementary file 2 [file DataSheet1.pdf]
